# Using Low Levels of Stochastic Vestibular Stimulation to Improve Balance Function

**DOI:** 10.1371/journal.pone.0136335

**Published:** 2015-08-21

**Authors:** Rahul Goel, Igor Kofman, Jerome Jeevarajan, Yiri De Dios, Helen S. Cohen, Jacob J. Bloomberg, Ajitkumar P. Mulavara

**Affiliations:** 1 Department of Health and Human Performance, University of Houston, Houston, Texas, United States of America; 2 Wyle Science, Technology and Engineering Group, Houston, Texas, United States of America; 3 Neuroscience Laboratory, NASA Johnson Space Center, Houston, Texas, United States of America; 4 Department of Otolaryngology–Head and Neck Surgery, Baylor College of Medicine, Houston, Texas, United States of America; 5 Universities Space Research Association, Houston, Texas, United States of America; McGill University, CANADA

## Abstract

Low-level stochastic vestibular stimulation (SVS) has been associated with improved postural responses in the medio-lateral (ML) direction, but its effect in improving balance function in both the ML and anterior-posterior (AP) directions has not been studied. In this series of studies, the efficacy of applying low amplitude SVS in 0–30 Hz range between the mastoids in the ML direction on improving cross-planar balance function was investigated. Forty-five (45) subjects stood on a compliant surface with their eyes closed and were instructed to maintain a stable upright stance. Measures of stability of the head, trunk, and whole body were quantified in ML, AP and combined APML directions. Results show that binaural bipolar SVS given in the ML direction significantly improved balance performance with the peak of optimal stimulus amplitude predominantly in the range of 100–500 μA for all the three directions, exhibiting stochastic resonance (SR) phenomenon. Objective perceptual and body motion thresholds as estimates of internal noise while subjects sat on a chair with their eyes closed and were given 1 Hz bipolar binaural sinusoidal electrical stimuli were also measured. In general, there was no significant difference between estimates of perceptual and body motion thresholds. The average optimal SVS amplitude that improved balance performance (peak SVS amplitude normalized to perceptual threshold) was estimated to be 46% in ML, 53% in AP, and 50% in APML directions. A miniature patch-type SVS device may be useful to improve balance function in people with disabilities due to aging, Parkinson’s disease or in astronauts returning from long-duration space flight.

## Introduction

Stochastic resonance (SR) is a phenomenon based on the concept of maximizing the flow of information through a non-linear system by the presence of non-zero level of noise [1–3]. Stochastic resonance has been shown to improve function in a variety of human physiological systems including motor, cardiovascular, visual, hearing, and balance function. Stochastic electrical stimulation of the vestibular system (stochastic vestibular stimulation, SVS), using SR principles, has been shown to improve heart rate responsiveness in healthy subjects [4], visual processing in healthy [5] as well as stroke patients [6, 7], cardiac and motor function in patients with central neurodegenerative disorders [8], and postural responses in patients with Parkinson’s disease (PD) [9, 10].

Over the past century the transcutaneous delivery of electric currents to the vestibular afferents commonly referred to as galvanic vestibular stimulation (GVS) has been used to study and understand the function of the vestibular system (for review see [11]). Bilateral bipolar vestibular electrical stimulation applied across the mastoid bones with a constant current profile has generally been shown to induce plane-specific body sway in the medio-lateral (ML) or anterior-posterior (AP) direction depending on if subject’s head is facing forward or if it is turned to the side (i.e., over the left or right shoulder), respectively [12, 13]. However, in contrast, stochastic vestibular stimulation (SVS) has been used to examine disruption of control of nominal body responses in posture, balance, and gait to unpredictable vestibular perturbations [12, 14, 15]. Studies have shown that using 3–5 mA peak binaural bipolar current levels of modified sum-of-sine’s current profile across the mastoid processes in the ML direction disrupted balance function in both ML and AP directions [16]. In our previous work, the application of low levels of bipolar SVS with peak currents in 100 to 400 μA range between the mastoids, on healthy subjects, while standing on an unstable surface, resulted in significant improvement of balance performance in the ML direction [17]. Other studies have investigated the potential of low levels of SVS to improve balance control in PD patients in both ML and AP directions. Pal’s study [9] found reduction of sway in the AP direction when SVS was applied to patients with PD during a balance task. In that study, the cathode was placed on both the mastoids and the anode was placed on the C7 vertebra, in a ‘bicathodal’ configuration, delivering stimulation to the vestibular end organs in the AP direction on both sides [9]. Samoudi’s study [10] showed similar improvement in balance control in PD patients with SVS application across the mastoids (ML direction) during recovery from perturbations in AP direction. Postural control in both ML and AP directions was improved with stochastic noise when applied as mechanical vibration to the soles of the feet [18, 19], or when applied as electrical noise to the knee [20] via SR phenomenon. Hence, one of the goals of this project was to investigate specific cross-planar improvements in balance control (in both ML and AP directions) given the SVS stimulation in the ML direction.

Intensities of electrical stimulation scaled to a fixed ratio of subjective thresholds used in previous studies showed improvement in physiologic functions with the SR phenomenon. Threshold to the imposed stimuli could be defined, as in previous studies, by examining physiologic responses (body motion response or eye movement response) or perceptual responses, based on subjects identifying what they feel (e.g., nociceptive sensation, virtual movement, nausea) either using a stochastic signal or a sinusoidal stimulus profile. Some studies have used electrical stimulus amplitudes that were chosen to be 90% of a physiologic threshold such as nystagmus [5] or 60% of nociceptive threshold [8] or 100% of body motion threshold to a 1 Hz sinusoidal stimulus [10]. Others have identified sensory threshold to the stimulus by gradually increasing the stimulus intensity (usually in steps of 0.02, 0.05, or 0.1 mA) until the subject reported tingling [5, 21, 22] or vestibular sensations [23]. One study even assumed a fixed threshold of 1 mA across all subjects [24]. Using a systematic approach to find an objective estimate of threshold will be useful not just in the context of SR but for other studies exploring the use of high amplitude levels of currents to disrupt balance function for simulating acute loss of vestibular function in general. Customizing the intensity of stimulus for SR applications scaled to an objective estimate of individual’s physiologic threshold will help to maximize improvement effects at sub or peri-threshold levels and also help to avoid using relatively large levels of stimulation that may elicit undesirable responses. Similar approach of using customized optimal amplitude of vibratory stimuli as a function of individual sensory threshold estimates have shown improved balance performance in subjects with functional ankle instability [25]. Therefore another goal of this project was to determine the optimal amplitude level of electrical SVS for improving balance function as a function of the estimated stimulus induced motion threshold.

## Materials and Methods

Data presented in this paper were collected during four different studies, on healthy subjects as shown in Table 1. To investigate cross-planar effects, we have included data from our previously published study that examined improvements only in the ML direction. In Study 1 [17], only a balance task was conducted and no thresholding data were measured from subjects. In Study 2, a balance task was conducted and the thresholding data were obtained following this on a different day. In Study 3, a thresholding task was conducted first, followed by a spatial orientation task (results will be part of another paper) on the same day. In Study 4, a thresholding task was conducted first, followed by the balance task on the same day.

**Table 1:** Information regarding stimuli, total number of subjects, number of responsive subjects in different directions for the different studies.

	Study 1	Study 2	Study 3	Study 4
**Order of Balance Task**	1	1	N/A	2
**Order of Thresholding Task**	N/A	2	1	1
**Peak of Bipolar Stochastic Stimulus Ranges in Balance Task**	0, 100, 200, 300, 400, 500, 700 μA	0, 100, 200, 300, 400, 500, 700, 900, 1100, 1300, 1500 μA	N/A	0, 20, 40, 60, 80, 100, 160, 220, 280, 340, 400% of perceptual threshold
**Peak of Bipolar Sinusoidal Stimulus Ranges in Thresholding Task (μA)**	N/A	100, 200, 300, 400, 500, 600, 700, 900, 1100, 1300, 1500	100, 200, 300, 400, 500, 600, 700, 900, 1100, 1300, 1500, 2000	100, 200, 300, 400, 500, 600, 700, 900, 1100, 1300, 1500, 2000
**Number of subjects**	15	15	11	15
**Number of SVS responsive Subjects in ML direction**	9	13	N/A	10
**Number of SVS responsive Subjects in AP direction**	9	11	N/A	10
**Number of SVS responsive Subjects in APML direction**	7	9	N/A	6
**Number of SVS responsive Subjects with Threshold data in ML direction**	N/A	12	N/A	10
**Number of SVS responsive Subjects with Threshold data in AP direction**	N/A	10	N/A	10
**Number of SVS responsive Subjects with Threshold data in APML direction**	N/A	9	N/A	6

### Participants

For the first three studies, 35 subjects were recruited from the Human Test Subject Facility at NASA—Johnson Space Center (JSC) in Houston, TX, USA. Study 1 and 2 used 15 healthy subjects each, and Study 3 used 11 healthy subjects, six of whom were repeat subjects from Study 2. All of these subjects had passed the equivalent of an Air Force Class III physical examination within 12 months of beginning the study. In Study 4, 15 healthy subjects were tested at Baylor College of Medicine (BCM). These 15 subjects underwent head-thrust, Dix-Hallpike, and vestibular evoked myogenic potential (VEMP) tests to screen them for any vestibular impairment. No subject had any reported history of otologic, neurologic, cardiovascular, orthopedic, or traumatic disorder. For the 50 subjects, mean ± standard deviation (SD) age was 34.5 ± 9.5 years, height was 173.9 ± 10.0 cm, and weight was 75.4 ± 14.3 kg, with normal or corrected-to-normal vision.

### Ethics Statement

The experimental protocols were approved by the Institutional Review Boards at NASA JSC and BCM. All subjects gave their written informed consent before participating in the study and were free to withdraw at any time.

### Procedures

#### Electrode Placement

For each subject, the 5 cm x 10 cm electrodes (Alexgaard Manufacturing, CA, USA) were centered over the mastoid processes on both sides using methods described in our previous paper and described briefly herein [17]. The skin surface at the electrode sites was cleaned and dried, and a layer of electrode gel was applied before placing the electrodes on the skin surface. Soft pads were then placed over the electrodes and held in place using an elastic strap that did not constrain head movements. This methodology was adopted to achieve a uniform current density and minimize any irritation at the electrode site during the delivery of the electrical stimulus to the skin. The impedance between the electrodes was confirmed to be less than 1 kΩ.

#### Balance Task

Balance performance was assessed in Studies 1, 2 and 4 (see Table 1). Subjects wore comfortable clothes and socks during all testing sessions. Subjects performed a standardized balance task of standing on a block of 10-cm-thick medium-density foam (Sunmate Foam, Dynamic Systems, Inc., Leicester, NC, USA), feet together, head facing forward, with their eyes closed and arms crossed in front of them for a total of up to 44 s per trial as described in our previous paper [17]. Balance performance was measured using a force plate (Kistler 9286B, Kistler, Amherst, NY) under the foam block and inertial measurement unit (IMU) motion sensors (MTx, Xsens North America Inc., Los Angeles, CA) placed on the head and trunk segments. Anti-slip tape was secured between the foam and the force plate to eliminate relative motion between them. A single data acquisition program (LabVIEW, National Instruments, Austin, TX, USA) collected time-synchronized data from the force plate and IMUs. The force plate data were filtered using an anti-aliasing filter implemented in the force plate signal processing hardware at 200 Hz before being sampled at 1,000 Hz. The IMU data were sampled at 100 Hz. If a subject opened his eyes, raised his foot, or spread his arms, then it was considered a “fall” and that trial was repeated a maximum of one time.

#### Thresholding Task

Threshold estimation was conducted in Studies 2, 3, and 4 (see Table 1). Subjects sat on a wooden stool with backrest, centered on a force plate, with their feet placed on the footrest, and held a gamepad (Logitech Gamepad F310, Lausanne, Switzerland) that had two joysticks. Subjects were facing forward with eyes closed. Subjects were instructed to report any perceived motion sensation by moving one of the joysticks with their dominant hand. The IMUs were attached to the head and trunk segments to record body motion. The exact instruction given to each subject was: “Use your dominant hand to push a joystick depending upon the direction of the motion sensation. Make sure to do it as long as you feel the sensation”. Data were collected synchronously from the IMUs at 100 Hz and from the joystick and force plate at 1000 Hz.

#### Subjective Assessment

Subjects were asked for feedback and comments after the tasks on any unpleasant sensation they might have felt during the tasks. In addition, in Study 4, subjects were asked to use the second joystick available on the gamepad with the non-dominant hand to report any non-motion sensation occurring during the thresholding task. The specific instruction given was: “Sometime, people can feel metallic taste in mouth, flashing in eyes, prickly or tingling sensation as well. Use the non-dominant hand to indicate these sensations by pulling the other joystick to either left or right and holding it until the sensation goes away”. Further, they were also asked to complete a simple questionnaire immediately after the thresholding and balance tasks, to track the presence and severity level of any unpleasant perceptions during testing (adapted from [26]). Subjects were asked to rate the severity on a scale varying from 1 (none) to 5 (very strong) on possible adverse effects: pain, tingling, itching, burning, vertigo, fatigue, nervousness, difficulty in concentration, changes in headache perception, unpleasantness or any visual sensations.

### Stimulation profile

During both tasks, a portable constant-current stimulator with subject isolation was used to deliver a bipolar constant current stimulus in the ±5 mA range to a load of up to 10 kΩ. The stimulator was powered using a 3.7 V battery pack and was operated in a standalone mode with the stimulation profiles stored on its internal flash memory card.

#### Balance Task

The duration of each balance trial was up to 44 s with two periods: baseline period lasting for up to the first 21.5 s had zero amplitude levels, while stimulus period spanning the remaining 22.5 s presented the signal at the selected amplitude level. Stimulus levels used in the different studies are presented in Table 1. Bipolar stochastic stimulation signals, similar to those used in our previous study [17] were generated using a white noise generator in the 0–30 Hz frequency range with peak stimulation levels ranging from 0 to 4000 μA, in steps of 50 μA. In our previous study, we applied a wide range of imperceptible noise levels to the vestibular system of subjects during a standing balance task and compared the effects of SVS on postural balance using both a colored noise signal in the narrow band 1–2 Hz and a white noise signal in the wide band 0–30 Hz frequency ranges [17]. The two signals were found to be similarly effective in improving balance through a reduction of sway. It was also reasoned with supporting evidence from [27] and [28] that the wideband range of 0–30 Hz improved performance by stimulating vestibular hair cells that affect posture (lower frequency sensitivity) and evoke vestibulo-myogenic response in the lower limbs (higher frequency sensitivity). Therefore, in this study a white noise signal in the wideband 0–30 Hz frequency range was used in the stimulation profile. The fully generated signal was checked for zero mean (±1%) and root mean square [(26 μA RMS/100 μA peak) ±5%]. The order of trials was randomized across subjects in each study. The trials with zero amplitude current during the stimulus periods were considered control trials while all other trials were the experimental trials.

#### Thresholding Task

The thresholding task was designed to identify the level of vestibular stimulation at which subjects were able to discern/or exhibit motion induced by the stimulation. Previously, in studies investigating effects of GVS, a periodic stimulus had been used to identify sensory threshold, which is usually the intensity at which the experimenter observed body sway at the input frequency [10, 12, 29]. In other studies a stochastic signal was used to define threshold based on subjects’ perception of irritation on the skin [8, 26]. Hence, in Study 2, two different types of stimulation signals were used for thresholding: 1 Hz sinusoidal signal and 0–30 Hz stochastic signal. The 0–30 Hz stochastic signal was the same as that used during the balance task. However, subjects reported perceiving no motion when the stochastic signal was used in the stimulation profile with current amplitudes between 0 and ±1.5 mA. This may be attributed to the stochastic nature of the electrical stimulation. Hence, the 1 Hz sinusoidal electrical stimulation signal was chosen for threshold determination and was used in all studies. Based on feedback from Study 2, before subjects performed the thresholding task in Studies 3 and 4, a familiarization trial was performed (using a short 38 s profile, 1 Hz sinusoidal signal, ±2 mA peak current) to make subjects aware of the kind of motion they may perceive during the thresholding task.

In general the stimulus profile consisted of 15 s periods with the 1 Hz sinusoidal stimulation signals, interspersed with 20 s periods of no stimulation. Based on feedback from Study 2, durations of baselines preceding the 15 s stimulation bursts were varied randomly in the range between 20 s and 23 s in Studies 3 and 4 in order to reduce the predictability of timing of the start of stimulation signals during the thresholding task. The different current peak-to-peak amplitude ranges used for Studies 2, 3 and 4 are shown in Table 1. A 2 mA peak current stimulus was added to the start of the profile used in Studies 3 and 4 so that the first stimulation level during the thresholding task was the same as what the subject had received during the familiarization trial. The order of the remaining stimulation levels, within the profile, was randomized and kept the same for all studies. The total duration for this task was 407 s in Study 2 and 463 s in Studies 3 and 4.

### Data Analysis

#### Balance Task

All data analyses were performed using MATLAB (MathWorks, Natick, MA) scripts and functions. The force plate and IMU data acquired for the middle 20 s during each of baseline and stimulus periods were first filtered at 10 Hz using a first order, zero phase response, low-pass Butterworth filter. The root mean squares (RMS) of 12 physiologic measures (six each in ML and AP direction) were calculated to characterize the balance performance of subjects during baseline and stimulus period for each of the 11 current ranges. The six balance parameters in the ML direction were RMS of: shear force (Fy), roll moment (Mx), linear accelerations for the head, and trunk segments (Hay, Tay), roll angular velocity for the head and trunk segments (Hrv, Trv) and the six balance parameters in the AP direction were RMS of: shear force (Fx), pitch moment (My), linear accelerations for the head and trunk segments (Hax, Tax), pitch angular velocity for the head and trunk segments (Hpv, Tpv). The first two parameters in each direction reflect postural responses across all segments of the subject and were calculated using the force plate data (global variables), and the remaining four parameters reflect the upper body segmental postural responses (local variables) and were calculated using the data from IMUs attached to the head and trunk segments.

Methods similar to those used in our previous paper [17] were used to evaluate improvements in balance performance. Thus, for each subject, the ratio of stimulus period RMS to the baseline period RMS for all experimental trials was normalized with that for the corresponding control trial, for each of the 12 parameters. A cost function was estimated as the sum of these ratios across all the relevant parameters for each direction (6 in case of ML and AP directions or 12 in case of APML direction) for all trials. Previous studies with application of SVS defined improvements in postural responses in PD patients by reduction in measures of sway in both AP and ML directions [9, 10]. Adding the variability in the global indicators of sway as measured from the force plate and local variables as indicated by the individual segmental IMU measurements would allow a more robust assessment of balance control. Hence, we used the minimum of the cost function as the determinant of improvement in performance. A number of studies that have shown improvement of function using the SR phenomenon have limited the magnitude of stimulus at or below the subject threshold ([5, 8, 10]). Further, other studies [30, 31] have shown that balance function can be improved by repeatedly exposing subjects to supra-threshold sum of sine electrical stimulation of the vestibular system by the process of desensitization of the sensorimotor integration process to ignore inputs from the vestibular system. Therefore, for each subject, the optimal stimulus amplitude (i.e., optimal trial) for the ML and AP directions, and across both directions combined (APML direction), were determined as the one at which the value of the cost function value was the lowest among the trials with non-zero stimulation periods with amplitudes less than the subjects threshold magnitude. We also identified subjects as responsive, i.e. exhibit improvement in balance performance in experimental trial compared to corresponding control trial, if the value of this cost function within any of their trials with stimulus amplitudes less than the subjects threshold magnitude was less than that calculated for their control trials.

We pooled together data for all parameters from the control and optimal trials for all subjects for further analysis. Data from all measures were then compared using multivariate repeated measures ANOVA. This analysis used all six measures for the ML and AP directions, and 12 measures for APML direction, with two within-subject factors: Period (2 levels—baseline and stimulus), and Trials (2 levels—control and optimal) at a significance level of 0.05. In addition, a paired t-test was used to compare performance, using the ratios of all parameters for stimulus with respect to baseline periods, between the control and optimal trials at a significance level of 0.05.

#### Thresholding Task

Raw joystick data were used in data analysis. For body motion measures, the data were first filtered at 1.5 Hz using a first order low-pass Butterworth filter and then at 0.5 Hz using a first order high-pass Butterworth filter, straddling the frequency of stimulation (1 Hz). This helped in eliminating some of the instrumentation noise, which is often present in physiologic measurements (e.g., sensor noise). Further, test subjects can also mask the effects of stimulation by being very fidgety (inadvertent movements not induced by stimuli during the trials) or stiff (resist imposed sway). To identify and screen out potential outliers, cross-correlation and frequency analyses were carried out on the body motion measures. For each stimulation period, cross-correlation functions were obtained between each of the six filtered body motion measures and the electrical stimulation profiles at the different amplitudes within each trial. Typically, any movement of the subject is expected to be captured by both the force plate and the IMUs. Therefore, if it was observed that the cross-correlation peak (within the first two cycles of stimulation period, at any stimulation level) for any body motion measure differed from other motion data, then the raw data for that measure were manually reviewed by the first and last authors. In the frequency domain, the dominant frequency was expected to be close to 1 Hz. If it was not, the raw data were manually reviewed to subjectively assess if the person was stiff or fidgety and if he/she was, then that data were discarded. During pilot testing for Study 2, subjects’ reports of perceptual responses of motion during applied stimulus were predominantly in the ML direction. Hence we limited our analyses of joystick and body motion to the ML direction only. The six measurements that characterized body motion in the ML direction were: (1) shear force (Fy); (2) roll moments (Mx); (3) linear acceleration for the head segment (Hay); (4) roll angular velocity for the head segment (Hrv); (5) linear acceleration for the trunk segment (Tay); and (6) roll angular velocity for the trunk segment (Trv). The first two body motion measurements were obtained using the force plate data and the remaining four body motion measurements were obtained from IMUs attached to the head and trunk segments.

#### Threshold Determination Algorithm

For the joystick data, percentage time of “perceived motion reported by the subject” for each stimulation and baseline periods was calculated. Joystick movement was interpreted as “perceived motion reported by the subject”, when its output amplitude exceeded 0.05 V (full-scale movement recorded in 0–5 V range). For the body motion data, for each of the six measures, the percentage time was estimated during each stimulation/baseline period for which body motion was detected by the sensors. Sensor data were interpreted as body motion whenever the amplitude exceeded 2 standard deviations from the mean calculated over the last 5 s of the baseline level before each stimulation level. The percentage time at each stimulation and baseline level for perceptual and body motion detection was normalized with respect to the largest value across all levels of stimulation. While the primary criterion was the decision whether the subject detected the signal or not, there were only two possible outcomes, “yes” (1) or “no” (0), hence a binomial distribution function was fit to the data with a generalized linear model (GLM) and a logit link function, which is very common in psychophysical studies [32]. This yielded a “maximum likelihood” model fit in terms of the probability of an event occurring (P(Y)) given by:

$$P(Y)=\frac{1}{1+e^{-(b_{0}+b_{1}X)}}$$

where *X* is the predictor variable (stimulation amplitude), and *b*_*0*_ and *b*_*1*_ are the coefficients of logistic regression. The data in this case were two MATLAB vectors–stimulation amplitudes (*X*) and the corresponding normalized percentage time of motion detection (*Y*). The function call in MATLAB (version R2011a with Statistics Toolbox v. 7.5) was [*b*, *dev*] = glmfit(*X*, *Y*,’binomial’,’logit’), where *b* is a two-element vector of regression coefficients (b_0_, b_1_ above), *dev* is the deviance (a generalization of the residual sum of squares) and is an indicator of goodness of fit. The threshold curve is generated using regression coefficients, by the function call glmval (*b*, *X*,‘logit’). Threshold was defined as the amplitude of stimulation at the point of subjective equality, at which there is a 50% chance of motion detection [2, 32]. *dev* > 4 was also used to screen for potential outliers in perceptual responses.

One of the goals was to compare subjects’ physiologic response threshold, obtained by measuring body motion (with IMUs and force plate), with the perceptual threshold based on joystick responses. There was no significant difference in thresholds for the six repeat subjects across Studies 2 and 3 (paired t-test, p = 0.5). Hence, we inferred that the small changes in procedures in threshold task during Studies 3 and 4 that were done based on feedback from Study 2 would not have affected their outcome. Therefore, threshold values obtained from perceptual and body motion measures were pooled together from subjects across studies 2, 3 and 4 for all further analyses. Thresholds obtained from the perceptual estimate (using the joystick) and the six motion measures (using the force plate and IMUs) were compared using repeated measures ANOVA with a significance level of 0.008 (after applying Bonferroni correction). We have also performed linear regressions between the perceptual measurement of threshold and physiological measurements of threshold to investigate how they are related with a significance level of 0.008 (after applying Bonferroni correction).

We were specifically interested in investigating the relationship between the perceptual threshold amplitude and the peak stimulation level that resulted in optimal balance performance from subjects participating in Studies 2 and 4 for whom both threshold and balance assessment data were available. Data from both studies were pooled together and used for further analysis. A linear regression analysis was performed to examine the relationship between the peak stimulus amplitude at which optimal balance performance was determined and perceptual threshold amplitude using a significance level of 0.05.

## Results

### Balance Task

**Fig 1:**
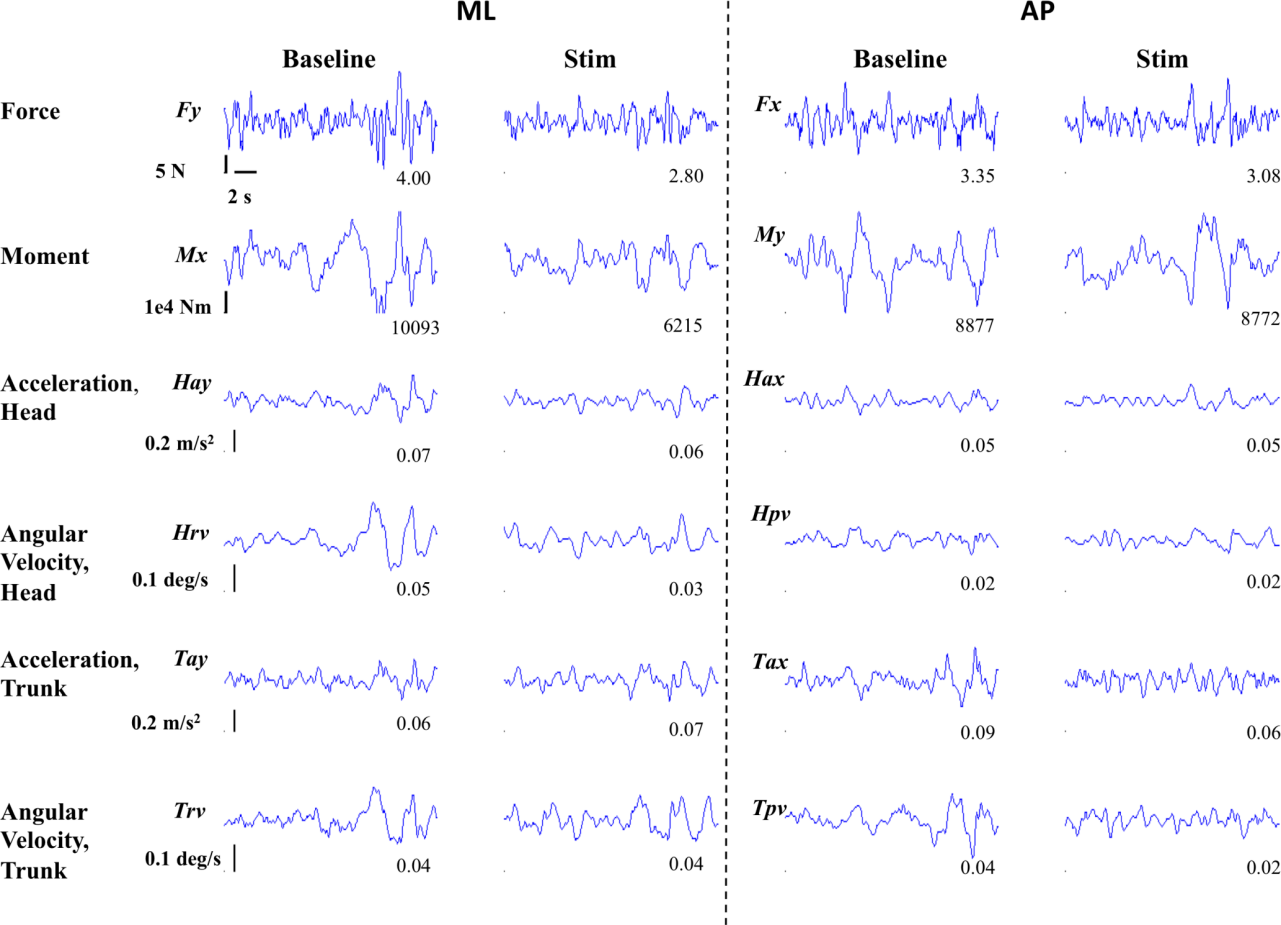
An exemplar plot of the 12 measures (ML direction: Fy, Mx, Hay, Hrv, Tay, Trv and AP direction: Fx, My, Hax, Hpv, Tax, Tpv) of interest for one subject during the balance task, for both baseline and stimulus periods for an optimal trial at the level of 350 μA (= 40% of this subjects perceptual threshold). Numerals in the bottom right of each panel represent RMS value.

**Fig 2:**
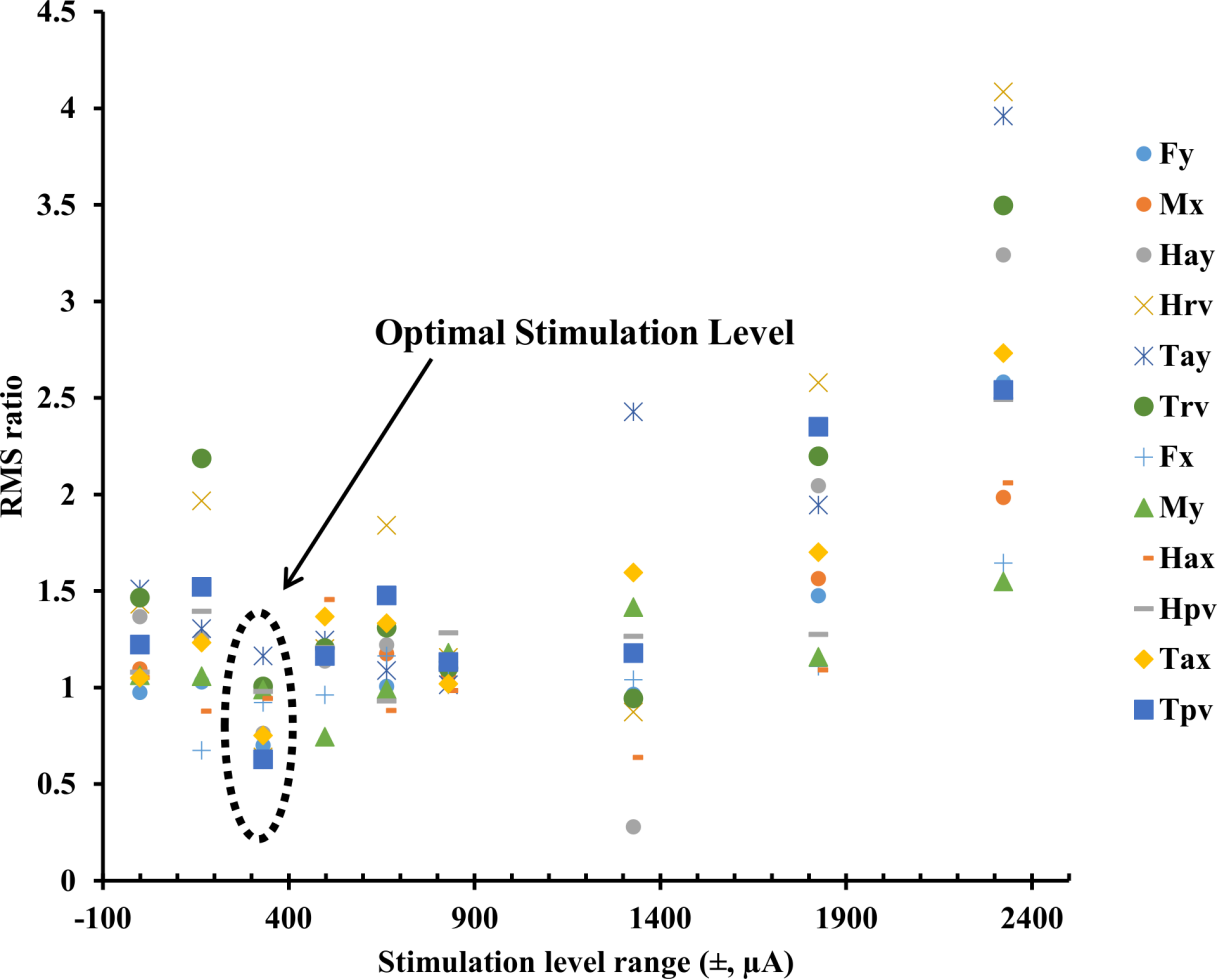
Ratio data of all twelve measures of RMS during the stimulus period to the RMS during the baseline period at different stimulus level ranges, for the same subject as in Fig 1. Note that for this subject, performance improved in all directions (ML, AP, and APML) at the same stimulus amplitude range of ±350 μA. Further, this subject had to be assisted to prevent falling and did not complete trials at the two highest nominal stimulation levels tested (±2800 μA, ±3300 μA).

Fig 1 shows an exemplar plot of all the measures (ML direction: Fy, Mx, Hay, Hrv, Tay, Trv and AP direction: Fx, My, Hax, Hpv, Tax, Tpv) for one subject during the balance task for both baseline (left columns) and stimulus (right columns) periods for an optimal trial at the stimulation level which had a peak current amplitude of 350 μA. For this subject, the RMS values (shown in lower right of each panel) for the stimulus period were less than or equal to those for the baseline period for all measures except Tay. Fig 2 shows the ratios of RMS during the stimulus periods to the RMS during the corresponding baseline periods for the same twelve parameters and for the same subject as in Fig 1. These data show that for this subject the optimal response in all directions was at the stimulation level which had peak current amplitude of 350 μA, which was approximately 40% of this subject’s perceptual threshold (830 μA). This subject completed all trials from 0% to 280% of his perceptual threshold and needed assistance to prevent fall (did not complete trials) at the two highest current levels tested (340% and 400%). Note that all subjects did not show optimal response at the same stimulus amplitude range for different directions (ML, AP and APML–see data in S1 Table).

**Fig 3:**
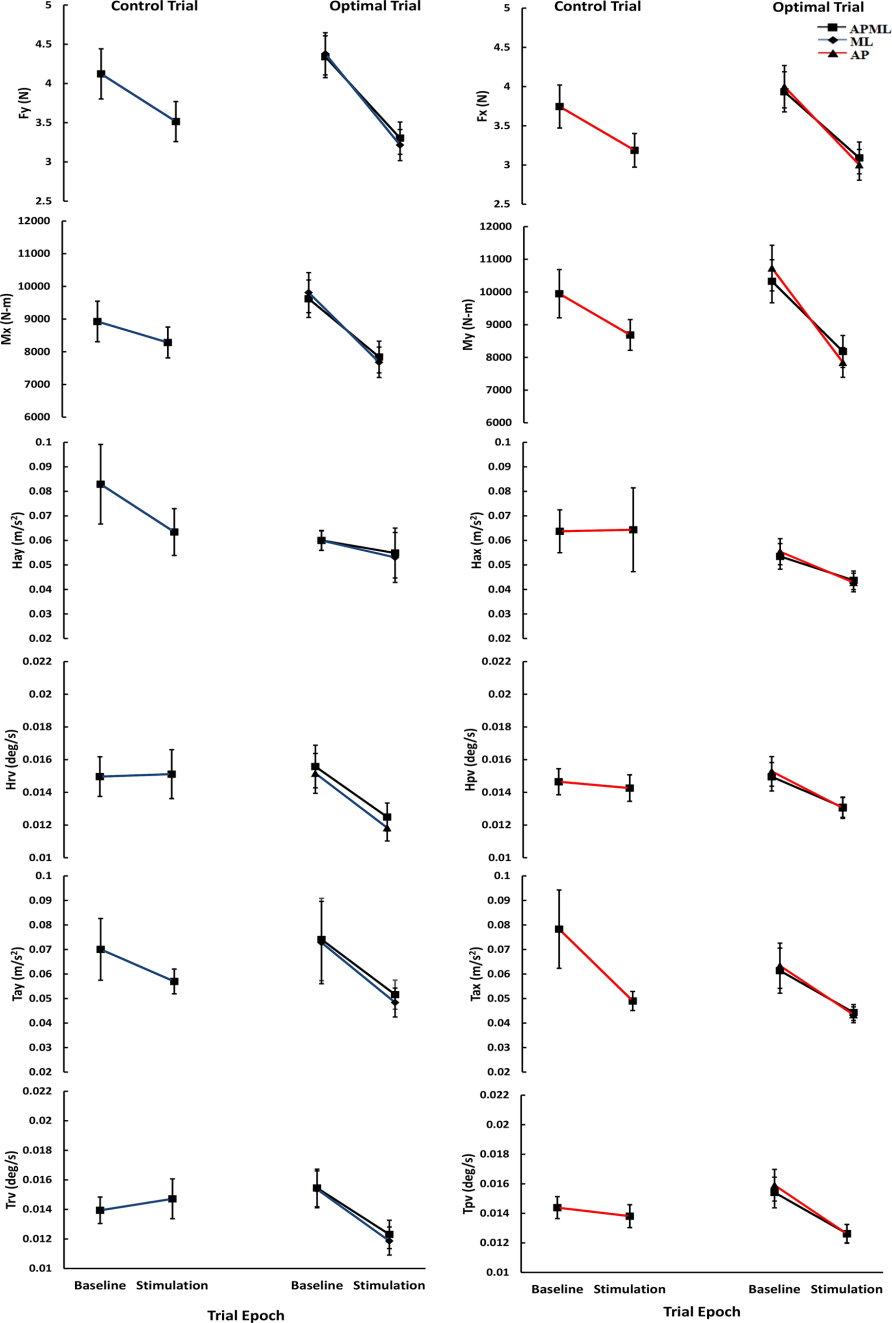
Mean (± one Standard Error of Mean) across all subjects (n = 45) showing RMS values of the six measures of interest for ML (see data in S2 Table), and AP (see data in S3 Table), and twelve measures for APML (see data in S4 Table), during the two periods (baseline and stimulus) of the control and optimal trials.

Fig 3 shows the mean (±one Standard Error of Mean (SEM)) across all subjects (n = 45) showing RMS values of the control and optimal trials for the six parameters of interest in the ML and AP directions, and for all twelve parameters for the combined APML direction. Fig 3 shows that for all the six parameters obtained from the ML or AP directions or all 12 parameters in the combined APML direction, the optimal stimulation trials compared with the no-stimulation control trials have average values that decreased by a greater magnitude during the stimulus period compared with the baseline period. Three separate repeated measures multivariate ANOVA were carried out, one each for ML, AP, and APML directions. All three showed that the within-subject factor, Period, was significant (ML: Wilks’ Lambda = 0.271, *p*<0.001; AP: Wilks’ Lambda = 0.390, *p*<0.001; APML: Wilks’ Lambda = 0.298, *p*<0.001), and the interaction Trial * Period was also significant (ML: Wilks’ Lambda = 0.599, *p* = 0.002; AP: Wilks’ Lambda = 0.722, *p* = 0.038; APML: Wilks’ Lambda = 0.554, *p* = 0.035). The significant interaction of Trial * Period is explained by the larger differences in RMS amplitudes for all the parameters between the baseline and stimulation periods for the optimal trials compared with that for the control trials (Fig 3). A paired t-test for each of the measures, comparing the ratios of parameter values for stimulus with respect to baseline periods between the control and optimal trials revealed that there was a significant difference between the trials for all of the six parameters in ML direction (Fy = 0.0005; Mx = 0.00005; Hay = 0.009; Hrv = 0.003;Tay = 0.0008; Trv = 0.0003); four of six parameters in AP direction (Fx = 0.007; My = 0.0004; Hax = 0.024; Hpv = 0.083; Tax = 0.086; Tpv = 0.002) and eight of twelve measures in the combined APML direction (Fy = 0.004; Mx = 0.001; Hay = 0.032; Hrv = 0.008; Tay = 0.008; Trv = 0.002; Fx = 0.059; My = 0.048; Hax = 0.088; Hpv = 0.131; Tax = 0.987; Tpv = 0.015). Overall, these results show that as a group normal healthy subjects significantly improved their balance performance at the optimal trials of SVS in comparison with control trials without SVS, thus, verifying and extending results from our previous study in a larger number of subjects and across planes [17].

Given these results it is important to point out that these analyses also showed that only a subset of all subjects, specifically thirty-two out of 45 subjects for the ML direction, 30 out of 45 subjects for the AP direction, and 22 out of 45 subjects for the APML direction, were responsive to the SVS, showing improvement in the optimal trials with respect to their respective control trials in balance performance. Two responsive subjects also showed greater improvement in the AP direction at supra-threshold current amplitudes (one at 127% and another at 154% of their perceptual thresholds). These two subjects also showed optimal improvement in APML at the two current levels as in the AP direction. In addition, one subject showed most improvement in APML direction at supra-threshold current amplitude (220%), but the second best improvement was at sub-threshold current amplitude (40%).

Table 2 shows mean (SEM) percentage improvement across all subjects responsive to SVS for different measures in the optimal stimulus trials during stimulus period normalized to baseline period value with respect to that for the control trial for uni-planar (ML or AP separately), and cross-planar (both ML and AP combined) analysis. Overall, for the responsive subjects across all parameters, SVS resulted in an average improvement in the range of 18% to 26%, 14% to 26%, and 16% to 29% in the optimal trials with respect to the control trials for ML, AP, and APML directions, respectively.

**Table 2:** Mean (SEM) percentage improvement across all subjects responsive to SVS for different measures in the optimal stimulus trials during stimulus period normalized to baseline period value with respect to that for the control trial for uni-planar (ML or AP separately), and cross-planar (both ML and AP combined) analysis.

	Uni-planar		Cross-planar	
Parameters	ML (n = 32)	AP (n = 30)	ML (n = 22)	AP (n = 22)
**Force**	18.7 (3.54)	21.9 (3.33)	20.9 (3.90)	23.1 (2.89)
**Moment**	22.2 (3.78)	26.2 (4.05)	23.5 (4.99)	17.9 (6.40)
**Head acceleration**	22.0 (3.66)	23.7 (4.32)	22.6 (4.54)	23.6 (3.15)
**Head velocity**	23.8 (3.95)	19.3 (4.01)	25.8 (5.04)	16.6 (4.81)
**Trunk acceleration**	22.9 (3.16)	14.2 (5.13)	21.3 (4.01)	19.8 (2.06)
**Trunk velocity**	26.7 (3.53)	18.5 (4.03)	29.6 (4.21)	19.7 (4.01)

### Thresholding Task

**Fig 4:**
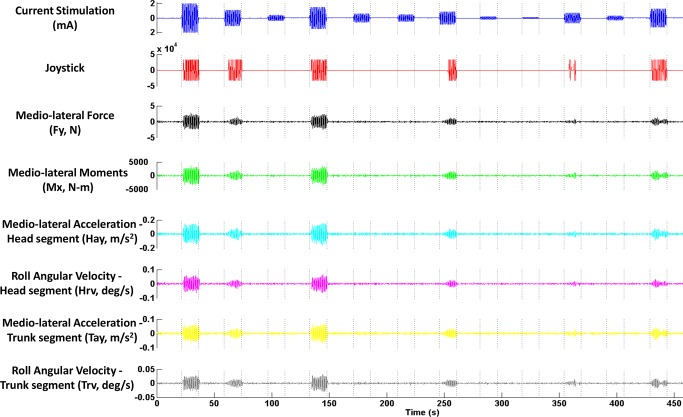
Exemplar plot of different measures of interest for a typical subject during the thresholding task.

**Fig 5:**
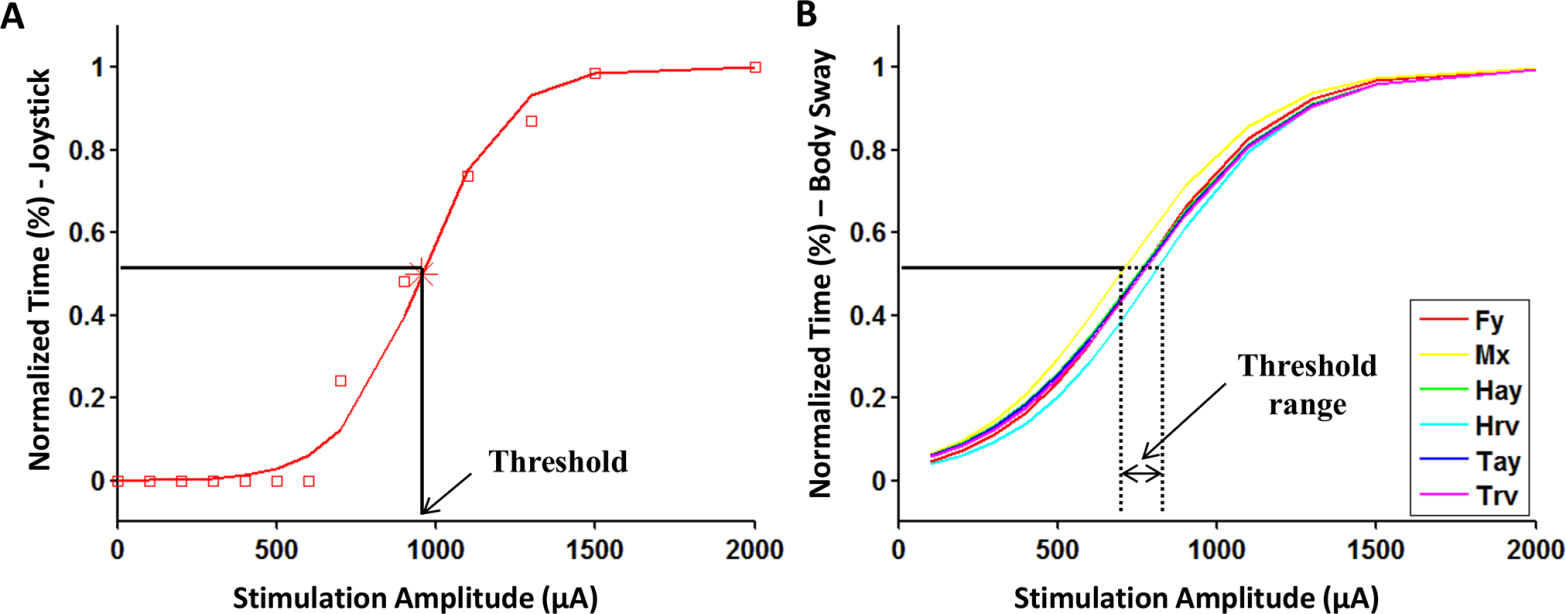
Nonlinear regression fitted lines of logistic psychometric function on the normalized percent time data at different stimulation amplitudes for a typical subject. (A) motion perception using Joystick data (red squares indicate normalized percent time of perceptual motion detected at each stimulation level), (B) body sway using physiological data: Fy, Mx, Hay, Hrv, Tay, Trv (individual normalized percent time data points quantified for each of the different body sway measures are not shown for clarity).

Fig 4 shows an exemplar plot of the measurements of interest for one subject during the thresholding task. Fig 5A shows the typical distribution of normalized percentage time of perceptual motion detected at each stimulation level indicated by red squares, for the same subject as in Fig 4. The solid red line, also called the threshold curve, indicates the non-linear regression fit obtained from the logistic function. Fig 5B show the threshold curves obtained using the six measurements of body motion (for the sake of clarity, the normalized percentage time of body motion for each measurement at different stimulation amplitudes is not shown). Threshold is the abscissa value at which the curve crosses the relative 0.5 probability of detection as indicated.

**Fig 6:**
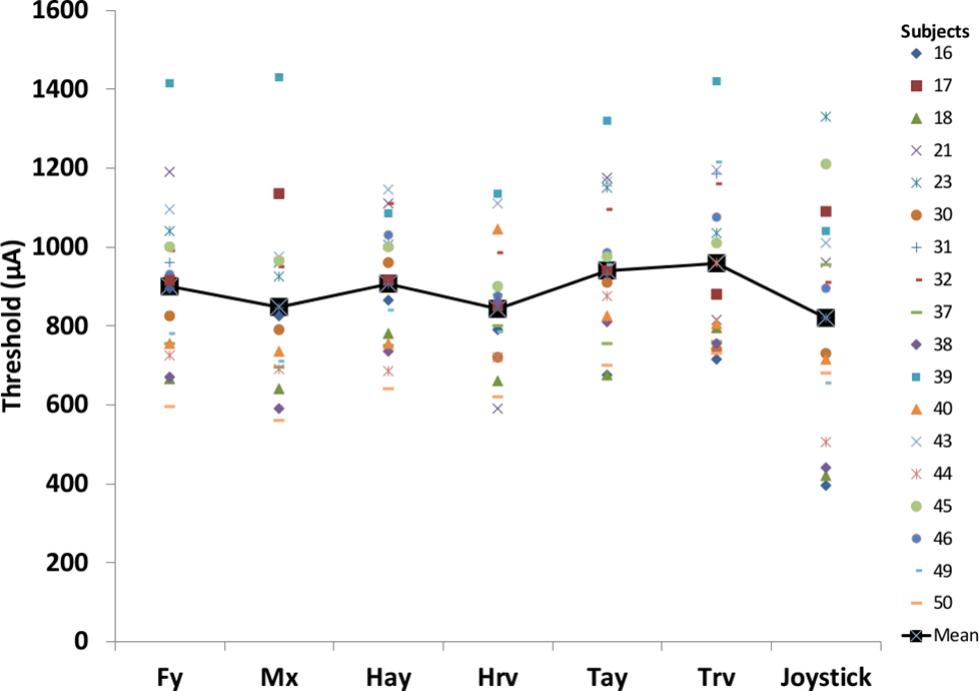
The threshold amplitude data estimated using the seven measures (perceptual: Joystick, and physiological body sway: Fy, Mx, Hay, Hrv, Tay, Trv) for each of the 18 subjects (see data in S5 Table).

The first and last author manually reviewed potential outliers suggested by the cross-correlation and frequency analyses of the body motion measurements and *dev* values for the curve fit for both body motion and perceptual motion detected across all subjects for all measurements and amplitude levels. For six subjects who were part of both Studies 2 and 3, thresholding data were used from Study 3 thus reducing the number of subjects’ data available for analyses to 35. One subject was removed as instructions were not followed by this subject and did not move the joystick during the thresholding task. Another subject was removed from the analysis because equipment noise persisted in the physiologic measures throughout the thresholding task. Furthermore, two subjects showed multiple false positives during the baseline periods (0 stimulation) of their perceptual motion detection responses. One subject was determined to be fidgety based on physiological measures and the other subject did not follow instructions. False positives cause the threshold curve to become too deviant and result in unreliable estimates of thresholds and hence these two subjects were not included in the analysis. Additionally, 13 subjects were removed from the analysis, as their physiologic measures were not consistent as they were either fidgety or stiff. Eight of these 13 subjects showed motion during baseline periods well above the baseline equipment noise variations (fidgety). For five out of these 13 subjects, no motion was detected by force plate or IMUs even though they indicated perceiving motion using the joystick input (stiff subjects). Fig 6 shows the threshold amplitude data estimated using the seven different measures–one measure of perceptual response and six measures of body motion, from each of the remaining 18 of the 35 subjects showing acceptable responses across all seven measures.

The repeated measures ANOVA showed that thresholds obtained using the seven measures were different from each other (F(3.5, 59.8) = 3.2, *p* = 0.024, Greenhouse–Geisser correction applied). Follow-up paired t-tests for each of the seven measures, revealed that there were no significant differences in thresholds obtained from perceptual measurements to the thresholds from any of the body motion measures after Bonferroni correction was applied (Fy: p = 0.126; Mx: p = 0.586; Hay: p = 0.100; Hrv: p = 0.709; Tay: p = 0.019; Trv: p = 0.039).

Table 3 shows the linear regression slope and intercept values between independent variable of perceptual thresholds from joystick measurements with the different dependent variables of threshold obtained using the different physiological measures. P-values are for the slope of the regression equation (n = 18). As seen from the regression analysis only the lateral force, moments and linear acceleration of the head and trunk segments show a significant relationship with the perceptual threshold obtained using the joystick measurement after Bonferroni correction was applied for multiple comparisons. Among the body motion measures, thresholds obtained from each of the two angular velocity measurements of the head and trunk segments (Hrv, Trv) did not show a significant relationship with the threshold determined using the joystick even though the paired t-tests showed no significant differences in the magnitudes of thresholds. Overall, it can be inferred that, in general, there was no difference between perceptual and physiologic thresholds.

**Table 3:** Parameters from regression between threshold amplitudes obtained using each of the physiological measures and that obtained from the joystick (x). P-values are for the slope of the regression equation (n = 18).

X	Equation	R^2^	r	p
**Fy**	0.4871x + 500.56	0.4169	0.646	0.004
**Mx**	0.5024x + 435.52	0.4240	0.651	0.003
**Hay**	0.3705x + 602.55	0.4085	0.639	0.004
**Hrv**	0.2165x + 665.51	0.1457	0.382	0.118
**Tay**	0.4772x + 548.38	0.4912	0.701	0.001
**Trv**	0.3426x + 677.41	0.1948	0.441	0.067

We were specifically interested in using the perceptual threshold amplitude for predicting the stimulation level that would result in optimal balance performance. We used the threshold estimates obtained from the perceptual measure to compare with the optimal trial peak current levels using available data from 28 of 30 subjects who completed their balance assessments from Studies 2 and 4 (threshold data were not obtained from the 15 subjects who participated in Study 1 and one subject in Study 2 did not follow the instructions during thresholding procedure and one subject in Study 4 gave erroneous readings). However, optimal trial peak current levels in ML, AP and APML directions showed no significant correlations with the perceptual threshold amplitude (p>0.05).

### Subjective Assessments

The mean (SD) subjective assessment scores across 15 subjects of Study 4, after completing the thresholding and balance tasks are shown in Table 4. During the thresholding task, four out of 15 subjects reported metallic taste (for 21 s, 28 s, 26 s, and 0.3 s cumulative across the entire thresholding task) and three reported tingling under the electrodes (for 47 s, 9 s, and 35 s cumulative across the entire thresholding task), during the stimulation. These sensations were experienced at stimulation levels higher than their perceptual thresholds and no one experienced these sensations during their optimal balance trials.

**Table 4:** Mean (SD) of subjective assessment scores after thresholding and balance tasks (n = 15).

Adverse effects	Mean (SD) intensity of adverse effects during the thresholding task	Mean (SD) intensity of adverse effects during the balance task
**Pain**	1.00 (0.0)	1.20 (0.4)
**Tingling**	1.47 (0.8)	1.60 (0.7)
**Itching**	1.07 (0.2)	1.17 (0.4)
**Burning**	1.17 (0.4)	1.23 (0.5)
**Dizziness**	2.32 (1.0)	1.82 (1.0)
**Fatigue**	1.00 (0.0)	1.20 (0.5)
**Nervousness**	1.13 (0.4)	1.20 (0.5)
**Difficulty in concentration**	1.20 (0.6)	1.13 (0.4)
**Headache**	1.13 (0.4)	1.23 (0.5)
**Unpleasantness**	1.27 (0.5)	1.50 (0.6)
**Visual sensations**	1.00 (0.0)	1.97 (1.0)

## Discussion

Our results show that low levels of binaural bipolar SVS applied in the ML direction, significantly improved balance performance consistent with the SR phenomenon previously observed, in normal healthy subjects, in ML, AP, and combined APML directions, respectively. The peak amplitude of optimal stimulus for improving balance performance was predominantly in the range of 100–500 μA across all the directions. These results confirm and extend the results obtained in our previous study of improving balance control in the ML direction [17].

Our results show that binaural bipolar 1 Hz sinusoidal electrical stimulation can be used to provide an objective estimate of the threshold for induced movement. Thresholds obtained using perceptual responses were not significantly different than those estimated using direct physiologic measures of induced movement responses. The average current level of SVS stimulation that resulted in improved balance performance in terms of percentage of perceptual thresholds was estimated to be 46% in ML, 53% in AP, and 50% in APML directions.

### Balance Task

Overall our data show that the application of low levels of SVS resulted in a significant improvement in cross planar balance performance at a non-zero noise amplitude. Further, it is also important to realize that only a subset of these subjects showed improved balance control during the specific sub-threshold amplitude of stimulation with respect to their nominal performance in the control trial on an unstable foam surface with their eyes closed as indicated by the reduction of sway in ML (32 of 45 subjects), AP (30 of 45 subjects), and APML (22 of 45 subjects) directions (See Table 1 for the number of subjects in each study which showed improved balance control). This suggests enhanced stability in both ML and AP directions while standing on unstable surface with eyes closed with the application of the optimal amplitudes of external white noise in the range of 0–30 Hz. Another study [16] showed increased postural sway in the ML and AP directions due to the application of higher current amplitudes of up to 5 mA in the ML direction. Five mA noisy current at low frequency (<1 Hz) might have completely deregulated veridical information regarding inertial space coming from the vestibular system, as suggested by a recent paper by the same group [30]. Our results show reductions in sway in both ML and AP directions at sub-threshold stimuli but degradation of performance at supra-threshold levels (see higher stimulation levels in Fig 2). This behavior of SVS during the balance task showing improved performance at non-zero low amplitude current levels, reaching peak in performance at non-zero optimal stimulus level and degraded performance at higher levels suggest SR type behavior [2, 3].

This series of studies used SVS signals in the range of 0–30 Hz to enable the enhancement of information transfer across the wide responsive frequency range exhibited by the vestibular spinal function [14, 29]. Furthermore, balance performance was measured during the baseline period—the period in which 0 mA of current were presented, paired with stimulus period—the period when different amplitudes of stimulation currents were applied to the vestibular system, in both the experimental designs. This helped track subjects’ performance during stimulus presentation relative to their baseline ability to control their balance on the challenging compliant surface with their eyes closed for each stimulus amplitude trial. This approach was adopted to take into account the possibility of nominal improvement as the subject may adapt to the foam surface thereby biasing the effects of SVS on balance responses over each trial and across repeated trials. The amplitude variation in the average estimates of parameter values across subjects (Fig 3) in transition from the baseline to stimulus periods during the control trials, when no current was presented for the entire duration of the trial, indicates the inherent variability of the balance performance across subjects as they get more practice and adapt to the support surface characteristics within the trial. Given these baseline variations, our results thus indicate that for the optimal trials, the amplitude of sway was significantly reduced during the stimulation period relative to the baseline period, when compared with control trials, as a result of the application of the electrical stimulation to the vestibular system.

The average percentage improvements (Table 2) were comparable across all subjects responsive to SVS for all the three directions. Thus, SVS in the ML direction improved performance in both ML and AP directions, by the same order of magnitude. Improvements in both ML and AP directions, using sub-threshold current amplitudes, suggest that the SR phenomenon may not only occur at the peripheral sensory levels but also at the CNS [33]. Kim’s group [21] have demonstrated linear effects of noisy SVS, at 90% of threshold, on brain rhythms as measured by EEG signals, 20–25 s after stimulation ceased. It is important to note that this study [21] showed their effects when current stimulation was removed, whereas our methodology employs low-level SVS and the effect is measured while the stimulation is active. Feusner’s study [34] used cranial electrical stimulation and showed that during stimulation, small perturbations in brain oscillations may have significant effects in normal resting state brain activity. Perhaps at the sub-threshold stimulus levels, SVS modulates the activity across the brain via enhanced information transfer and may explain the observed improvement in balance control. Additionally, due to the biomechanics of the body, some researchers have suggested considerable amount of coherency between ML and AP directions [12]. Pavlik’s study [12] also found significant coherency between AP sway and input stimulus (applied in the ML direction) in few subjects. Hence improvements in ML direction can improve performance in AP direction as well.

Only 16 of 45 subjects showed optimization at the same current level in all three directions (see data in S1 Table). For 18 of 45 subjects, the optimal current level in ML was less than that in AP, whereas for a relatively equal number (11 of 45) of subjects, the optimal current level in AP was less than that in ML. Further, assessing the trials over which subjects showed improvements across 12 measures in the APML direction, 16 of 45 subjects showed optimal performance for the same trials as in the AP direction alone and 12 of 45 subjects showed optimal performance for the same trials as in the ML direction alone. Two subjects showed optimal performance at different stimulation levels in the three directions. It should be noted that in some previous studies showing improvement of postural response in both ML and AP directions using mechanical vibration [18, 19] researchers had used ML and AP measures separately. In contrast, in this study, we compared RMS of twelve measures of body motion to find the optimal in combined APML direction. Therefore, given the cross coupling effect for balance control and the above results, application of SVS in the ML direction may be sufficient for eliciting overall improved balance performance.

### Thresholding Task

One of the objectives in this project was to be able to customize subjects’ optimal stimulation levels as a function of their objectively measured stimulation-induced motion threshold. This approach can maximize improvements in balance performance effects for individual subjects as compared to other approaches such as using peri-threshold levels of stimulation as used in the Samoudi study [10].

We propose an objective method to determine a robust estimate of subjects’ motion threshold with a relative probability of detecting motion at 50% due to application of the 1 Hz sinusoidal electrical stimulation across the vestibular end organs. Bilateral bipolar GVS produces acceleration signal towards the cathode electrode, and body’s response/tilt is in the opposite direction, i.e. towards the anode electrode [11]. Similar to our design, others have also used electrical stimulation using sinusoidal waveforms in the frequency range of 1–2 Hz to determine the threshold by visually observing the amplitude at which subjects swayed at input frequency [10, 12]. These studies have used subjects’ motion threshold levels as the maximum amplitude limit during the posture task in which stochastic stimuli were used. Also, for threshold determination tests we used sinusoidal electrical stimuli while stochastic signals were used in other studies [8, 26] because the majority of our subjects did not discern the direction of induced motion during the application of stochastic stimulation (during pilot testing for Study 2). Stochastic nature of electrical stimulation of the vestibular organs may have precluded the possibility to objectively detect sway in one direction or the other. Further, sinusoidal motion are easy to discern and hence can yield responses that can be quantified.

One of the limitations of this technique is that because of the highly predictable nature of the sinusoidal stimulation it is possible that reported results might be influenced by learning or adaptive physiologic mechanisms. To avoid or reduce that effect, the order of presentation of stimulus amplitude was randomized and the 20 s of baseline period was extended by a variable time of 0–3 s in the last two studies.

We compared two types of responses: (1) perceptual responses reported by the subject focusing on motion perceived due to the stimuli indicated using a joystick, and (2) body motion that is induced in response to the stimuli that was measured using force plate and IMUs fixed to the head and trunk segments. 6% of the subjects (2 out of 35) had to be dropped because they erroneously reported motion sensation using the joystick at multiple times during baseline periods, which overestimated their threshold. One subject was deemed fidgety from the analysis of body motion sway measurements while another moved the joystick in accordance with the instructions to report sensations other than motion and hence was deemed as not following operator instructions. Measurement of any physiologic response is also masked by various sources of noise [33, 35, 36]. One subject was dropped because of higher noise levels in the physiologic motion measurement during threshold testing that was not exhibited during subsequent balance assessment. Additionally, body motion induced in response to the stimuli can be modulated by the subject if they are very stiff and resisting motion or if the subject is fidgety and is having difficulty sitting still during the trial. Parameters from coherence, cross-covariance, frequency response functions and cross-spectral measures that have been used in some other vestibular stimulation studies as well [12, 37, 38] to quantify relationship between the applied stimulus and the observed response were used in our analyses to identify stiff and fidgety subject body motion responses. 35% of subjects (13 out of 35) had to be dropped because of inconsistencies in their cross-correlation or frequency responses across the different body motion measures for a given stimulus amplitude. However, in general, there were no differences in the magnitudes of thresholds obtained from perceptual and body motion measures suggesting that subjects were correctly indicating when they were swaying. However there was a lack of linear relationship between perceptual threshold and specifically the thresholds obtained using measurements of angular velocity of both the head and trunk segments. This specifically indicates that thresholds determined using the angular velocity measurements might be less sensitive indicators as compared to that determined using the linear acceleration measurements from the IMU’s. Body motion measurements obtained with force plates, IMUs and other analog hardware have such disadvantages as susceptibility to instrumentation and environment noise, hardware costs, and dependence on subjects’ ability to remain still and be responsive during testing. Results of this study suggest that estimating thresholds from the subjects’ perceptual response (thus eliminating the aforementioned drawbacks) may be sufficient for various types of SVS applications.

### Application of Noise to Improve Balance Function

Some researchers are of the opinion that binaural electrical stimulation applied across the mastoids primarily activates semicircular canals [38], whereas some others believe that this type of stimulation primarily activates the otolith system [39]. The exact mechanism by which electrical stimulation applied across the mastoids affects balance function is still unclear but it is possible that both canal and otolith behavioral responses are generated and many factors like stimulus intensity, placement of electrodes, measurement etc. can affect what response occurs and to what extent [40]. GVS was hypothesized to probably act at the spike trigger zone of vestibular afferents [41, 42], rather than causing membrane depolarization: maintained GVS generated a maintained series of action potentials (which adapt) during the DC stimulus [43]. GVS activates the primary vestibular afferents, primarily via the irregular afferents, which have a lower threshold and are more sensitive to GVS than regular primary afferents [42, 43]). However, other studies have also demonstrated that electrical current limited to low frequency of stimulation applied across isolated Type I mammalian vestibular hair cells (VHC) evoked mechanical responses of fast length changes of the cell “neck” [44, 45]. These authors hypothesized that as mammalian VHCs are firmly joined to adjacent structures, such responses to imposed electrical fields may result in a change in the rotational mechanical characteristics of the steriocilia that will cause a modification in the hair bundle position and have a subsequent effect on the transduction process transfer function during head acceleration and tilt. Hence, we hypothesize that low levels of SVS may help to improve signal detection and information transfer based on the phenomenon of SR with both of these mechanisms at the vestibular hair cell or spike trigger zones of vestibular afferents as being potential causative factors. Improving signal detection or enhancing information transfer via the vestibular system using the SR phenomenon is further useful because of the convergence and modulation of its activity via proprioceptive inputs at the vestibular nuclei [46]. Further, the vestibular nuclei project to many areas in the central nervous system (CNS) including the flocculus of the cerebellum, the spinal cord, the oculomotor nuclei, and thalamocortical pathway ([11, 46]. One of those pathways may be involved in alteration of activity in the substantia-nigrae pars reticularis (SNr), as increases in γ-amino-butyric acid (GABA) release in the SNr can result from SVS [47]. A recent study, the first to explore the neurochemical effects of vestibular stimulation in hemi-Parkinsonian rats *in vivo*, evaluated motor effects of SVS at or below threshold levels [47]. That study, demonstrated corresponding improvements in behavioral responses in locomotor activity and reduced Parkinsonian symptoms [47]. SVS induced differential effects in the two substantia-nigrae (SN) with increased GABA release on the lesioned but not the intact side. In normal rats SVS stimulations increased GABA release in the intact SN, but not in other investigated areas. Thus, SR phenomenon with SVS may not only help enhance information transfer to occur at the peripheral sensory levels but also shows effects at other levels in the CNS [33]. SVS may help improve automatic postural control by also facilitating the vestibular spinal control system or other non-dopaminergic pathways as shown in PD patients [10]. Interestingly, it was recently reported that high-frequency deep brain stimulation (DBS) of the SNr improved axial symptoms and gait, when combined with DBS in the sub-thalamic nucleus in PD subjects [48]. It was hypothesized that SVS and DBS-SNr may in part act through a common mechanism by inhibiting the over-active SNr in PD patients [49].

For bidirectional stimuli, threshold can be considered as a measure of internal noise which includes all physiologic sources of variability (afferent noise, processing noise, etc.) [35]. Some investigators have hypothesized that the optimization of behavioral responses by externally applied noise may result from interaction between the applied external noise and the nominal internal noise present in the CNS [50,51]. Thus, if the internal noise level is high, the external noise required to elicit optimal response will be less. However we did not see evidence for this in our data. This may be the case as our perceptual threshold values may not be reflecting the true level of internal noise that may be estimated using more conventional methods such as that used by Merfeld et al. [35]. A histogram analysis of the optimal stimulation values do indicate that approximately 80% of subjects showed optimal stimulation values in the range of 100–500 μA across the ML, AP and APML directions (see data in S6 Table) while the perceptual threshold values are uniformly distributed in the range from 400 to 1400 μA. This shows that although the estimates of internal noise estimated using our threshold task methodology showed considerable variability across subjects the absolute amplitude of peak external noise that can improve balance performance in both ML and AP directions is over a relative narrow band of 100 to 500 μA.

Roughly 30% of our subjects (13 of 45 in ML and 15 of 45 in AP) did not show improvements in their optimal trials to SVS relative to their control trials with the application of low-levels of SVS in the ML direction. We have perceptual thresholds for nearly half of the non-responsive subjects and they were in the same range as those in responsive subjects, which indicates that internal noise levels were comparable across both responders and non-responders. We hypothesize that these non-responders may have preferences for inherently reducing the weighting afforded to the vestibular signals while increasing weighting of sensory contributions from vision and proprioception in the sensorimotor integration process for postural control [52, 53]. The demonstration in our data that two subjects improved balance in the AP direction at supra-threshold levels offer support to sensory reweighting by down-weighting vestibular information [30].

### Subjective Symptoms to Vestibular Stimulation

Dizziness, tingling, metallic taste and light flashes were the most common subjective symptoms reported by the subjects during the thresholding and balance tasks. These symptoms occurred at supra-threshold levels of electrical stimulations and thus it is unlikely that it would have affected subjects’ perceptual thresholds or the improvements in balance assessments. Most importantly, the stimulus levels during the optimal trials were sub-threshold and were not perceived by subjects. Lack of any major subjective symptoms is consistent with another study [26], which used stimulus amplitude up to 1.5 mA, and reassures that subjects were comfortable with sub threshold levels of SVS and it does not generate any unpleasant symptoms [10].

## Conclusions

To conclude, we have shown that an imperceptibly low level of white noise-based stochastic electrical stimulation of the vestibular system improved balance performance in both ML and AP directions, and is consistent with the SR phenomenon in normal healthy control subjects. In general, using stimulation amplitudes at 46% to 53% of perceptual motion threshold improved balance performance significantly compared to control (no stimulation) conditions. The peak amplitude of vestibular stimulation that improved balance function was predominantly in the range of ±100 to ±500 μA for all three directions. These results are consistent with the findings in our previous study [17]. We have also shown that sinusoidal electrical stimulation of the vestibular system can be used to objectively estimate subjects’ perceptual threshold for motion detection. Thresholds obtained using perceptual responses and the physiological measures were not different, however, there is higher cost involved with obtaining thresholds using physiological measures.

Higher levels of electrical stimulation have been shown to degrade postural performance by creating temporary vestibular disturbance to simulate clinical behavior or as a training tool [16]. Some researchers [30] have suggested repeated exposures to higher levels of electrical stimulation as a pre-flight training tool for astronauts to learn to down-weight vestibular information. In contrast, our methodology of using sub-threshold electrical stimulation will help retain and enhance the use of vestibular information in performance of specific functions. A device using electrical stimulation of the vestibular system based on SR principles may be useful as a miniature patch-type stimulator that can be worn by people with disabilities due to aging or vestibular associated movement disorders to improve posture, locomotion or movement function. It may also be useful as a training modality for astronauts to enhance adaptability or skill acquisition by increasing utilization of vestibular information and therefore will help us to optimize and personalize a sensorimotor adaptability countermeasure prescription. This may help to significantly reduce the number of days required to recover functional performance to preflight levels after long-duration spaceflight.

## Supporting Information

S1 Table Threshold vs Optimal. Page shows the Perceptual threshold (μA), optimal Amplitude Range (±, μA), the optimal amplitude range as a percentage of threshold (%), trial numbers at which the optimal stimulus trial was performed, and responsiveness of subject (Y) showing their optimal trial cost function value was less than that for their control trial across the three ML, AP and APML directions.
(XLSX)

S2 Table ML Optimal. This page shows the RMS values for the six variables Fy, Mx, Hay, Hrv, Tay, and Trv for the baseline and stimulation periods for the control trials and optimal trials for all subjects in ML direction.
(XLSX)

S3 Table AP Optimal. This page shows the RMS values for the variables Fx, My, Hax, Hpv, Tax, and Tpv for the baseline and stimulation periods for the control trials and optimal trials for all subjects in AP direction.
(XLSX)

S4 Table APML Optimal. This page shows the RMS values for the variables Fy, Mx, Hay, Hrv, Tay, Trv, Fx, My, Hax, Hpv, Tax, and Tpv for the baseline and stimulation periods for the control trials and optimal trials for all subjects in APML direction.
(XLSX)

S5 Table Threshold Data. This page shows the threshold amplitude determined using the physiologic (body motion) variables and the perceptual (joystick) variable for the subjects whose data were available for analysis. This page also shows the reason for subjects whose data became unavailable for analyses.
(XLSX)

S6 Table Histograms. This page shows the histogram analyses for the optimal trial stimulation amplitude (μA) in the ML, AP and APML directions.
(XLSX)
